# When they don't come around: how athletes and DCOs perceive the legitimacy of a remote sampling system in anti-doping testing

**DOI:** 10.3389/fspor.2026.1825917

**Published:** 2026-06-30

**Authors:** Daniel Westmattelmann, Benedikt Stoffers, Julian Lanfer, Andrea Petróczi

**Affiliations:** 1 Center for Management, University of Münster, Münster, Germany; 2 School for Business Administration, PHWT Vechta, Vechta, Germany; 3 Faculty of Health and Sport Sciences, Széchenyi István University, Győr, Hungary

**Keywords:** anti-doping, athletes, doping control, extended valence framework, legitimacy, remote sampling, technology acceptance, trust

## Abstract

**Introduction:**

Remote Sampling Systems (RSS) represent a technological innovation to traditional anti-doping testing, yet successful implementation depends on stakeholder acceptance. To move RSS beyond emergency use, system design must account for user acceptance. Drawing on a contextualized Extended Valence Framework (EVF), this study examines how athletes and Doping Control Officers (DCOs) evaluate RSS legitimacy and how benefit, risk, and trust perceptions shape their attitudes toward RSS introduction.

**Methods:**

A cross-sectional online survey with 132 athletes and 107 DCOs compared both groups on transparency, trust, perceived benefits, three risk dimensions (performance, privacy, psychological), perceived legitimacy, and attitude toward RSS. Group-specific PLS-SEM tested the hypothetical structural model, and multi-group analysis (MGA) identified significant between-group differences.

**Results:**

DCOs reported higher performance risk. Athletes showed higher legitimacy perceptions and more favorable attitudes. PLS-SEM explained substantial variance in legitimacy (63.8% athletes, 56.6% DCOs) and attitude (74.2% athletes, 56.1% DCOs). Transparency was positively associated with trust, which related positively to perceived benefits and reduced all risk dimensions. Perceived benefits, performance risk, and psychological risk shaped legitimacy, which was strongly associated with attitudes. MGA showed that transparency was more strongly associated with trust among DCOs, while performance risk and legitimacy showed stronger associations among athletes.

**Discussion:**

Perceived legitimacy emerges as the central mechanism linking benefit-risk evaluations to attitudes in a mandatory anti-doping technology context. Both groups converge on many RSS evaluations but diverge on performance risk and legitimacy. DCOs prioritize functional robustness; athletes are more receptive but sensitive to fairness. Findings highlight the need to address DCOs’ performance concerns and build legitimacy through transparent procedures, user-centered design, and communication that emphasizes fairness and effectiveness.

## 1 Introduction

Anti-doping testing constitutes a fundamental pillar of global sports governance, safeguarding both the integrity of competition and the rights and welfare of (clean) athletes. To ensure fairness and prevent the use of prohibited substances or methods, organizations with responsibility for anti-doping (NADOs, sport federations, and major event organizers) allocate considerable resources to implementing out-of-competition testing regimes (1, 103), which rely on the collection and analysis of biological samples from athletes, namely urine or blood samples (2, 103). Alternative minimally invasive matrices, such as dried blood spots (DBS), offer logistical flexibility and are therefore increasingly popular (3, 4). All of these testing programs are central to upholding the principles of fair play, serve as a deterrent against doping practices, and are intended to foster trust in athletic performance and competition outcomes (5–7).

The COVID-19 pandemic, however, posed unprecedented challenges to the anti-doping system, which became visible in the drop in test numbers in 2020 (8). Lockdowns and social distancing measures resulted in a significant disruption to in-person testing, revealing the vulnerability of traditional approaches that depend on physical interaction between athletes and doping control officers [DCOs (3)]. In response, several national anti-doping agencies launched pilot initiatives to explore Remote Sampling Systems (RSS) as an innovative solution to ensure the continuity of doping control (9, 10). These systems allowed for the remote supervision of sample collection, primarily through video communication tools and digital documentation, while maintaining compliance with the procedural standards of the World Anti-Doping Agency (4, 11).

At its core, remote testing involves the coordination between athletes and DCOs via secure digital platforms, through which sample collection, most commonly DBS or urine, is initiated, observed, and documented. Although the procedural frameworks and technologies supporting RSS application have matured, questions remain regarding its legitimacy, particularly in a field where sample integrity, chain of custody, and procedural compliance are critical (12). Viewed from a regulatory perspective, WADA currently allows application of remote sampling procedures only in the case of pandemic situations, when imposed contact restrictions hinder in-person testing (2). Taking the organizational perspective of ADOs as strategic actors in the anti-doping system, Stoffers et al. (11) find that interviewed ADO representatives are favorable towards wider application of RSS beyond pandemic situations: While a variety of risks are perceived, such as malfunctions of the RSS software or legal uncertainties in connection with remotely collected samples, the financial and ecological benefits of remote sampling procedures compared to in-person are widely acknowledged. Further pilot projects conducted by ADOs underline the abovementioned benefits of remote sampling, yet emphasizing the need for strict integrity requirements regarding the chain-of-custody of collected anti-doping samples, which becomes especially relevant in sight of doping criminalization and associated legal requirements (3, 13). Also, clarity regarding prerequisites to achieve athletes technological and procedural readiness with remote DBS procedures is emphasized (4).

These findings illustrate that for the successful implementation of such systems, it is not sufficient to consider technological and economic feasibility or regulatory endorsement alone. Rather, the perceptions of those directly involved in the testing process, namely athletes and DCOs, are central to evaluating the legitimacy and acceptability of remote testing. Their willingness to engage with these systems, their trust in the procedures, and their perceived risks and benefits are likely to influence both the uptake and effectiveness of RSS in practice (11). Technology adoption research supports this notion, emphasizing user perception as a key factor for successfully implementing new technologies in existing organizational processes (14, 15). In the context of anti-doping, perceived legitimacy of a rule or procedure is therefore a fitting critical lens to understand whether and how RSS can be integrated sustainably into testing programs with stringent integrity requirements (16).

While previous research has predominantly focused on organizational-level perspectives (3, 11) and the athlete experience with RSS has only been investigated in pilot settings (4), a research gap remains regarding a systematic assessment of the system's direct users, comprising not only athletes, but also DCOs. Our paper addresses this gap by specifically investigating and comparing the perspectives of both athletes and DCOs as the actual users of an RSS. Thus, the study aims to answer the following research question (RQ):

RQ: How do athletes and doping control officers evaluate the implementation of a Remote Sampling System, and how do key antecedents shape their perceptions of legitimacy and attitude towards a Remote Sampling System?

To address this RQ, the study adopts a legitimacy lens and applies a contextualized Extended Valence Framework [EVF (11, 17, 18)]. From a theoretical perspective, we reconceptualize EVF for a mandatory compliance setting by positioning perceived legitimacy, rather than mere usage intention, as the central evaluative outcome of a regulatory technology, and by linking perceived benefits, multiple risk dimensions, and uncertainty to both legitimacy and attitude. We further adapt EVF to the digital anti-doping context by incorporating anti-doping–relevant benefit and risk perceptions. We then empirically compare the legitimacy formation process across athletes and DCOs using structural equation modeling and multi-group analysis. This comparative perspective aims to explore where transparency, trust, perceived benefits, and risk perceptions exert similar effects on legitimacy and attitude, and where they differ between user groups, thereby advancing understanding of legitimacy formation in testing regimes with stringent integrity requirements.

## 2 Related literature on (remote) anti-doping testing

Doping represents one of the most significant threats to the integrity of professional sports (19–21). Aiming to detect doping and deter doping behavior in the first place by upholding the threat of severe doping sanctions, anti-doping testing follows the dual objectives of detection and deterrence (22). In this regard, streams of research focus on, e.g., enhancements in substance detection through testing [see e.g. (23, 24)], or on athleteś behaviors and predictors of doping intentions with associated incentives, and deterrent perceptions [see e.g. (25–30)]. The procedures to conduct anti-doping testing have remained largely unchanged for the past years. Apart from selected areas of process digitalization, such as the introduction of the anti-doping administration and management system ADAMS (31), testing still relies on in-person sample collection conducted by DCOs, who are trained to instruct and supervise the strictly regulated sample collection procedures (2).

**Figure 1:**
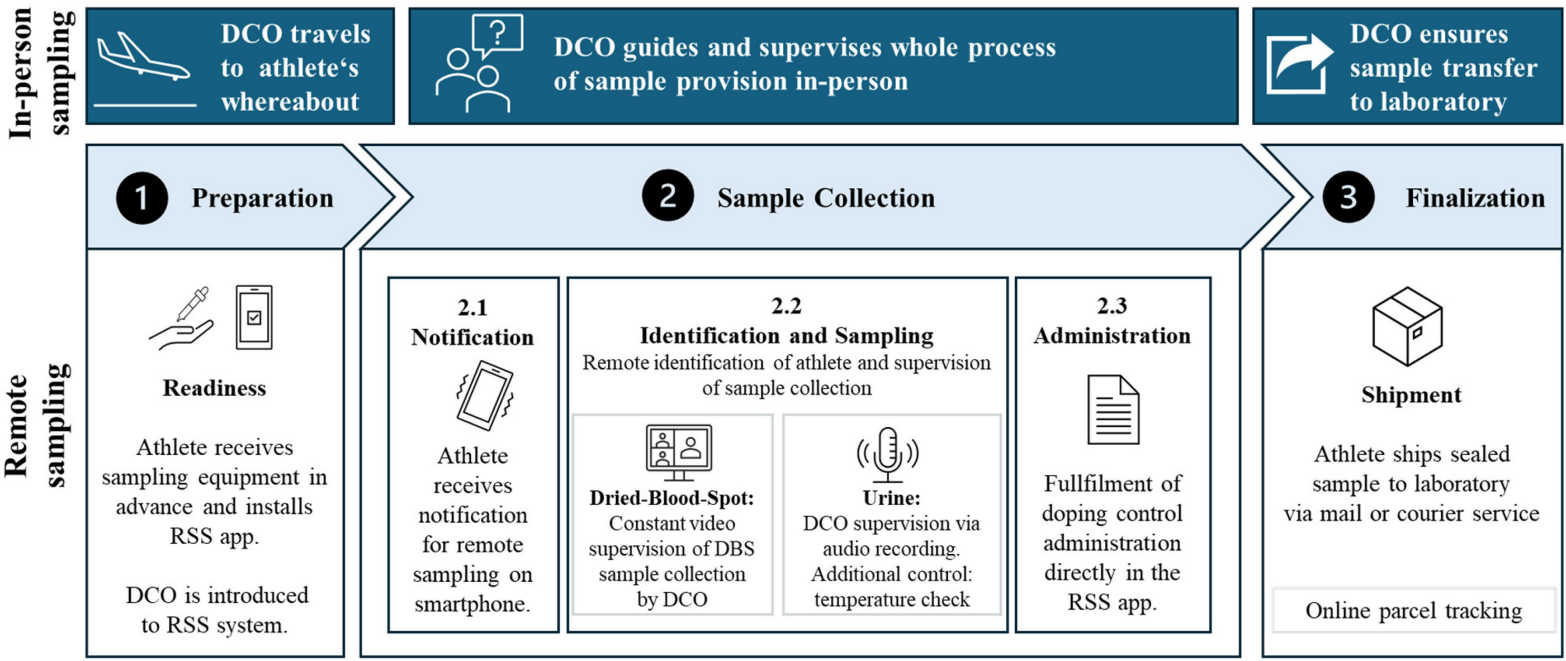
Remote sampling process. In accordance with Lanfer et al. (107), Stoffers et al. (11) and Trinks et al. (4).

As remote testing, a technological innovation of testing regimes, emerged during the COVID-19 pandemic, research in this specific area of anti-doping remains limited. Initial pilot programs, however, suggest that remote testing can be a viable supplement to traditional procedures (9, 10). USADA's virtual drug testing initiative was among the first to demonstrate that sample collection via video supervision is both technically and logistically feasible (10). Athletes self-collected urine and DBS samples using microsampling devices like the TASSO system, while trained personnel monitored the process remotely to ensure compliance with anti-doping standards (3). Expanding on this, Trinks et al. (4) evaluated a remote DBS testing system developed by NADA Germany in collaboration with Sportradar. Their dedicated, app-based “Remote Testing System” solution (32) integrates multifactor authentication and dedicated user interfaces for DCOs and athletes, enabling real-time video calls to supervise sample collection. Results showed the RSS and associated procedures were feasible and well-accepted by athletes. 24 elite athletes provided 102 DBS samples during the six-month pilot, all shipments reached the WADA-accredited laboratory, athletes rated handling as easy, and viewed remote testing as a complementary option. However, key constraints identified were connectivity concerns, customs liabilities that limit remote testing abroad, and availability issues. The remote sampling process, which was followed using the piloted RSS, is shown and compared to the traditional sampling process in Figure 1.

Such a process can be divided into three essential phases. In the preparation phase, technological and procedural readiness to enable remote sample collection is prepared. Specifically, the sample collection kit is delivered in advance to the athlete's location, and the athlete's availability for no-advance-notice sample collection is ensured through introduction to the associated remote testing smartphone application. The sample collection phase begins with digital notification by the DCO, followed by mutual identity verification and real-time supervision via video call. DBS samples are self-collected by the athlete using a device such as the TASSO (3, 33), while urine samples are audio-monitored with privacy safeguards and include a temperature check (9, 10). The finalization phase includes digital documentation, sealing of the sample, and tracked shipment to the laboratory, ensuring compliance with chain-of-custody standards (2, 32).

While the technical feasibility of remote procedures has been demonstrated, broader implementation depends on acceptance by multiple stakeholder groups: regulators, ADOs strategically planning testing regimes on an organizational level, as well as DCOs and athletes as users of an RSS. Stoffers et al. (11) addressed the regulatory and organizational stakeholder levels by qualitatively analyzing their perceptions of acceptance of implementing an RSS, using the Extended Valence Framework [EVF (17)] as a theoretical lens. Their study identified a generally positive attitude toward RSS among strategic and operational decision-makers. Benefits such as cost savings, increased testing flexibility, and reduced environmental impact were widely acknowledged. However, significant concerns were raised at the regulatory level, where skepticism centered on privacy, sample security, and the legal robustness of remotely collected evidence. Trust emerged as a critical factor influencing both perceived benefits and risk tolerance. The authors decomposed the core constructs of the EVF, perceived risks, perceived benefits, and trust, into subdimensions specific to the context of anti-doping testing, enabling a better reflection of the anti-doping integrity-sensitive nature. These included privacy risk, performance risk, and trust in system operators and technology providers.

Although these studies provide important insights into the potential and limitations of an RSS in anti-doping work, a crucial perspective remains underexplored: the views of the actual RSS users who are actively involved in the entire remote sampling process (see Figure 1). With regard to a sustainable and successful implementation of an RSS, it is essential to assess the perceived legitimacy and general attitude of introducing such a system from the athletes and DCOs perspectives themselves.

## 3 Theoretical Lens

We adopt the lens of legitimacy to examine how athletes and DCOs perceive the implementation of an RSS in anti-doping testing. In the anti-doping context, legitimacy is typically understood as perceived legitimacy, emphasizing that athletes and DCOs must believe in the fairness and appropriateness of anti-doping measures for those measures to be effective (34). The dimension *appropriate* refers to the perceived effectiveness of a measure, such as the RSS, in enhancing the effectiveness of anti-doping testing regimes (6, 35). *Proper* implies that institutions embody values shared by those affected and are viewed as eligible to define rules and procedures (18). Finally, *just* refers to the fairness of processes and procedures, including equal treatment and respectful sanctioning (18, 34). Given that perceived legitimacy is a psychological construct that drives subjective acceptance of new technologies rather than objective legitimacy according to laws or formal criteria (36, 37), the successful implementation of an RSS depends on whether athletes and DCOs, as RSS users, view it as *appropriate*, *proper*, and *just*. Thus, legitimacy offers a suitable theoretical lens for understanding users’ perceptions of adopting an RSS.

## 4 Research model and hypotheses

**Figure 2:**
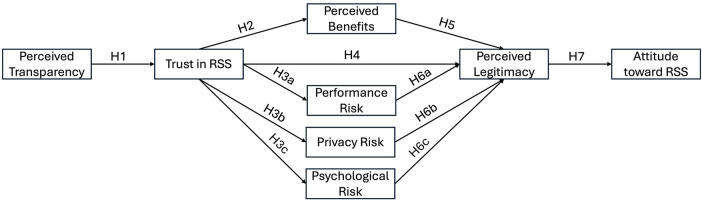
Proposed research model, H, hypothesis.

Taking the legitimacy lens, we developed the research model for this study (see Figure 2). To do so, we referred to the decomposed and contextualized EVF provided by Stoffers et al. (11) and set perceived legitimacy and attitude toward RSS as dependent variables. The EVF builds on the concept of valence to explain why and how people accept or reject technologies. Specifically, the EVF combines trust perceptions, perceived benefits, perceived risks, and a dependent variable such as attitude toward using an information system.

In this study, we further extend the EVF with the concept of perceived transparency when introducing an RSS (38) and derive the research model incrementally.

### 4.1 Perceived transparency

Perceived transparency, defined as “the perceived quality of intentionally shared information from a sender” [ (38), p. 5], is widely recognized as a critical foundation for trust across organizational [see e.g. (39)], governmental [see e.g. (40)], and digital technology contexts [see e.g. (41)]. In anti-doping, transparency has been linked to increased public support, perceived legitimacy, and athlete trust in regulatory systems (34, 42, 43). Transparent communication about procedures, rules, and data handling fosters understanding and reduces uncertainty, conditions under which trust is more likely to form (44, 45).

With the introduction of an RSS, which handles sensitive athlete data and marks a fundamental technological and procedural shift in how doping controls are conducted, transparency becomes increasingly relevant. Studies indicate that athletes and stakeholders value transparent processes when evaluating the legitimacy of innovations in anti-doping [e.g. (31)]. When users perceive that authorities (i.e., ADOs) openly communicate how the RSS operates, how data is secured, and how procedures are implemented, they are more likely to regard the system as trustworthy. This aligns with findings in information systems and organizational research that link transparency with enhanced trust in technologies and institutions (38, 46, 47). Accordingly, we propose:

H1: Perceived transparency of an RSS is positively related to trust in the RSS.

### 4.2 Trust in RSS

Trust, as defined by Mayer et al. [(48), p. 712], reflects “the willingness of a party to be vulnerable to the actions of another party based on the expectation that the other will perform a particular action important to the trustor, irrespective of the ability to monitor or control that party”. In technology adoption research, trust is frequently examined either in relation to the organization providing the technology (49, 50) or the technology itself (51, 52). This study follows the latter approach, operationalizing trust in RSS based on McKnight et al. (45).

The relationship between trust and perceived benefits has been substantiated in prior research. Kim et al. (17) demonstrated that trust exerts a strong positive influence on perceived benefits in the context of online purchasing. When users believe a system is trustworthy, i.e., that it is reliable, capable of functioning properly, and offers support, they are more likely to perceive its use as beneficial. This is especially pertinent in novel settings, such as remote testing, where prior user experience is limited and uncertainty is high (11). Therefore, the following hypothesis is proposed:

H2: Trust in RSS is positively related to perceived benefits.

Furthermore, empirical research supports the negative relationship between trust and risk perceptions in various domains, including e-commerce (53, 54), e-government (55), self-service technologies (52), and more recently, artificial intelligence services (49, 50). Specifically, trust has been found to lower performance-related concerns, privacy anxieties, and psychological discomfort associated with novel digital systems (17, 56). In the context of RSS, where athletes and DCOs may experience uncertainty regarding the system's accuracy, data protection, or psychological burden, trust serves as a stabilizing mechanism that mitigates these risk perceptions (11). Therefore, the following hypotheses are proposed:

H3a: Trust in RSS is negatively related to performance risk.

H3b: Trust in RSS is negatively related to privacy risk.

H3c: Trust in RSS is negatively related to psychological risk.

Moreover, several studies highlight a close connection between trust and legitimacy, particularly in the anti-doping domain. Trust contributes to the perception that organizations act properly and make decisions aligned with athletes’ interests, dimensions central to legitimacy (5, 18, 57). Conversely, distrust has been identified as a threat to legitimacy, undermining individuals’ willingness to accept institutional authority (58). As trust reduces concerns about effectiveness and fairness, it is likely to foster perceptions of legitimacy in novel anti-doping technologies like RSS. Based on this reasoning, the following hypothesis is proposed:

H4: Trust in RSS is positively related to perceived legitimacy.

### 4.3 Perceived benefits and risks

Perceived benefits contribute to legitimacy by signaling that the RSS is effective, productive, and meaningful in fulfilling anti-doping objectives (11). When individuals believe that a system enhances the efficiency, accessibility, or timeliness of anti-doping testing, they are more inclined to perceive it as legitimate (17, 59). This argument is supported by Henning and Dimeo (35), who emphasize that the perceived functionality and effectiveness of anti-doping measures are essential for their perceived legitimacy. Similarly, Overbye (6) finds that athletes who view testing measures as useful are more likely to accept and support them.

In contrast, performance risk may harm legitimacy by raising doubts about the system's reliability and functionality. When users believe that the RSS may produce incorrect or inconsistent outcomes, or that it might malfunction, they are likely to question whether the system is capable of fulfilling its regulatory purpose (17, 53). In the anti-doping context, this concern is especially salient, as any lack of accuracy may lead to unjust outcomes (e.g., false accusations or missed detections), thereby undermining athletes’ confidence in the system's fairness and effectiveness (11, 35). Prior research has confirmed that perceptions of functional inadequacy or system failure reduce the perceived legitimacy of regulatory systems (57).

Privacy risk further erodes legitimacy. If athletes feel that the RSS invades their private lives in ways that are disproportionate to its benefits, they may view it as illegitimate (34, 58). These concerns have been voiced frequently in both public discourse and empirical research on anti-doping, with athletes expressing discomfort over intrusive monitoring and excessive control (5, 6). Privacy violations are particularly problematic for legitimacy because they challenge fundamental norms of autonomy and dignity, which are central to athletes’ acceptance of institutional authority (18, 60).

Also, psychological risk, like the fear of emotional distress, anxiety, or perceived psychological pressure, may also negatively influence perceived legitimacy (11). If the use of RSS induces stress or a sense of constant surveillance, it may lead users to view the system as burdensome or even harmful, thus challenging its fairness and appropriateness (34). Because legitimacy depends on the perception that a measure is both necessary and fair, excessive psychological burden may suggest that the ‘costs’ outweigh the benefits, thereby diminishing the system's moral and procedural standing. This aligns with prior work highlighting the importance of subjective well-being and emotional responses in shaping legitimacy perceptions (18, 57).

Taken together, these arguments support the role of both positive (benefits) and negative (risks) system evaluations in forming legitimacy judgments. While perceived benefits enhance legitimacy, perceived risks undermine legitimacy. Therefore, the following hypotheses are proposed:

H5: Perceived benefits are positively related to perceived legitimacy.

H6a: Performance risk is negatively related to perceived legitimacy.

H6b: Privacy risk is negatively related to perceived legitimacy.

H6c: Psychological risk is negatively related to perceived legitimacy.

### 4.4 Attitude toward remote sampling system as dependent variable

In the anti-doping context, several studies emphasize the influence of perceived legitimacy on how athletes and DCOs evaluate testing procedures and related systems (58, 61, 62). While some work suggests that attitude can influence legitimacy (35), there is stronger empirical support for the reverse direction, that legitimacy perceptions shape attitudes [see e.g. (62),]. Recent empirical evidence supports this direction across diverse athlete populations and operationalizations. García-Grimau et al. (63) report a direct positive association between legitimacy and attitudes toward doping in Spanish elite track-and-field athletes within an extended Sport Drug Control Model specification. Barkoukis et al. (64) show that legitimacy perceptions exert both direct and indirect effects on anti-doping policy support, with the indirect pathway running through social-cognitive variables such as attitudes, norms, and intentions. Building on this, Barkoukis et al. (65) provide qualitative evidence on how athletes translate legitimacy perceptions into behavioral support for anti-doping policies. Clancy et al. (66) further differentiate between legitimacy of rules and principles on the one hand and legitimacy of authorities on the other, suggesting that these two dimensions may operate through partly distinct mechanisms.

Although perceived legitimacy and attitude are conceptually adjacent, they capture theoretically distinct evaluations. Perceived legitimacy reflects a normative judgment about whether the system is appropriate, justified, and fair within the rules and goals of clean sport (18, 37). Attitude toward RSS, by contrast, captures a summative individual evaluation of introducing the system along an evaluative dimension (67, 68). Within our research model, legitimacy operates as the cognitive-normative mediator that channels benefit-risk evaluations into attitudinal outcomes. The two constructs are therefore conceptually adjacent but not interchangeable. An individual may consider the system legitimate in the abstract while holding mixed personal attitudes toward its actual introduction, and conversely, a strongly unfavorable attitude need not imply a denial of legitimacy. Preserving this distinction allows us to test whether legitimacy can be built through transparency, trust, and balanced benefit-risk perceptions independently of the resulting attitudinal evaluation.

Moreover, the EVF (17), which underpins this study's research model, supports the view that evaluations of system fairness, purposefulness, and alignment with individual or group values inform users’ attitudes toward adoption. These legitimacy-related judgments help form an initial cognitive appraisal, which subsequently shapes the overall affective response toward a system. This has also been confirmed in related studies in the anti-doping field, where legitimacy has been shown to contribute to support for or opposition to doping controls (34, 35). Therefore, attitude towards RSS is the dependent variable in our model and the following hypothesis is proposed:

H7: Perceived legitimacy is positively related to attitude toward RSS.

## 5 Method

### 5.1 Research context

This study is part of a larger project (“Implementing a remote sampling system in anti-doping work”) funded by the Partnership for Clean Competition (PCC), which investigates the multi-stakeholder perceptions of RSS in anti-doping. To provide a comprehensive understanding of this technological shift, the project was designed in two complementary phases. The first phase, a qualitative exploration, utilized semi-structured interviews to identify the “lived realities,” core concerns, and perceptions among athletes and DCOs (107). These qualitative insights, detailed in a companion paper, informed the development of the conceptual model tested in this study. This current study represents the second phase, providing a quantitative validation of those factors to statistically compare how transparency, trust, and various risk dimensions influence the perceived legitimacy and overall attitude toward RSS across both user groups.

### 5.2 Research design and participants

To test the proposed research model (see Figure 2), a web-based survey was designed and translated into German, English, Spanish, Danish, Norwegian, Russian, Serbian, and Kazakh to facilitate broad stakeholder inclusion and accommodate the global reach of the participating partner organizations. Two separate links to the surveys, one for athletes and one for DCOs, were distributed via several international partner organizations (e.g., international sports federations or ADOs) to ensure that each user group received the survey designated for them (see Table 1 for demographics). A power analysis determined the required sample size for testing the research model, targeting an effect size of 0.4, with *α* = 0.05 and powe*r* = 0.9, in a model comprising eight latent and 32 observed variables. This analysis yielded a minimum sample size of 100 (69). We assumed a large effect size (> 0.4) in the power analysis because the dominant relationships in technology-acceptance PLS-SEM models of the type tested here consistently yield large effects in published meta-analytic and primary studies, particularly for the legitimacy–attitude and trust–risk perception relationships (70, 71). Our observed effect sizes for the central paths confirm this assumption ex post. We acknowledge that small-to-medium effects may be underpowered in the DCO subsample, which is addressed in the Limitations section.

**Table 1:** Demographics.

Variable	Athletes	DCOs
*N*	132	107
Age	Mean: 25.9 years (SD = 5.9)	Mean: 49.7 years (SD = 13.4)
Gender	43.2% female	38.3% female
Experience with remote testing	5.3%	5.6%

### 5.3 Measurements

To ensure a robust assessment of the research model, only established and validated scales were used to measure the variables (see Table 2). These original scales were carefully adapted to the specific context of remote sampling. All original scales, except for attitude, are 7-point Likert scales with answer options ranging from 1 (“strongly disagree”) to 7 (“strongly agree”). Attitude was measured using opposite pairs (e.g., extremely negative/positive, extremely pleasant/unpleasant) on a five-point bipolar semantic-differential scale, following Karahanna et al. (68). Where established scales used a 7-point Likert format, we retained the original anchoring. Where the canonical operationalization uses a 5-point semantic differential [Attitude toward RSS, following (68)], we retained that scale as published to preserve psychometric comparability with prior validation work. As PLS-SEM internally standardizes all indicators, differences in original scale range do not affect parameter estimation (72), and no additional standardization was applied.

**Table 2:** Measurements.

Construct	Source	# of items	Operational definition
Attitude toward RSS	Karahanna et al. (68)	4 (one dropped*)	The overall assessment of the potential introduction of the system.
Benefits	Kim et al. (17)	5	The perception that utilizing the RSS is more convenient, time-saving, and efficient than conventional testing, leading to increased productivity and reduced financial costs.
Legitimacy	Petróczi and Woolway (87)	4	The evaluation of the RSS as a fair, effective, and fully justified anti-doping tool that enhances equality across different sports and countries while protecting the integrity of clean sport.
Performance Risk	Featherman and Pavlou (53)	5 (one dropped*)	The perceived likelihood that the RSS or its supporting servers will malfunction, process information incorrectly, or fail to meet the expected level of service performance required for a successful doping control.
Privacy Risk	Rauschnabel (104)	5	The degree of concern regarding the potential for the RSS to collect excessive information, fail to protect user privacy, or allow personal data to be accessed by unknown parties or misused during the sampling process.
Psychological Risk	Sun (105); Casidy and Wymer (102)	3	The risk that the requirement to use the RSS will result in a negative emotional state, specifically causing users to experience unnecessary tension, unwanted anxiety, or a high level of worry.
Transparency	Venkatesh et al. (91) Welch et al. (106)	4 (one dropped*)	The belief that testing authorities provide deep access to the operational working processes of the RSS and offer genuine opportunities for users to provide feedback on the system.
Trust in RSS	McKnight et al. (45)	11 (six dropped*)	The user's expectation that the RSS is a reliable, dependable, and functional piece of software that will not fail or malfunction, and which provides competent, effective guidance or help when needed.

*Item(s) excluded due to low factor loadings (see Table 3).

### 5.4 Data analysis

We analyzed the data in two stages. First, prior to testing the research model, we compared group means for all latent constructs. Independent-samples Welch's *t*-tests were conducted in IBM SPSS Statistics v30 (73). To control error from multiple comparisons, we applied the Holm–Bonferroni correction (74). Mean differences were evaluated at the 5% level of significance (*p* < 0.05), and effect sizes were reported as Cohen's d (75).

Second, we estimated the research model and hypotheses using partial least squares structural equation modeling (PLS-SEM). Analyses were run in SmartPLS 4.1.1.4 (76) with bias-corrected bootstrapping based on 5,000 resamples, conducted separately for athletes and DCOs (77). PLS-SEM was preferred over covariance-based SEM because it accommodates complex structural models and does not assume multivariate normality (77, 78). Because established scales were adapted to the unique context of remote sampling, we followed a rigorous two-step evaluation process to ensure the instruments’ validity before testing structural paths. Step one involved evaluating the measurement model to assess indicator quality, reliability, and validity. Internal consistency reliability was evaluated using Cronbach's Alpha (ɑ) and Composite Reliability (CR), which measures the overall reliability of a collection of heterogeneous but similar items. While ɑ provides a lower bound of reliability, CR is calculated using the item loadings and provides a more accurate estimate in PLS-SEM. We also confirmed convergent validity—the extent to which a measure correlates positively with alternative measures of the same construct—using the Average Variance Extracted (AVE). Discriminant validity was assessed to ensure constructs are conceptually distinct from one another, utilizing the Fornell-Larcker criterion and the Heterotrait-Monotrait (HTMT) ratio. Step two consisted of estimating the structural model to evaluate the hypothesized relationships. The exploratory nature of our model further supports the choice of PLS-SEM (77). Moreover, our sample sizes (*N* = 132 athletes; *N* = 107 DCOs) and constructs measured with more than two indicators mitigate potential PLS-SEM biases discussed in the literature (77). Finally, bootstrapping enhances the robustness of PLS-SEM estimates (77).

Beyond estimating the model for each group, we conducted a multi-group analysis (MGA) to test for significant differences in path coefficients between athletes and DCOs (72).

To assess potential common method bias (CMB), we conducted two complementary tests. First, following Kock (79), we computed full collinearity variance inflation factors (VIFs) by regressing each latent variable on all others using SmartPLS 4. Full-collinearity VIF values were mostly below the conservative 3.3 threshold. The exceptions were Attitude in the DCO sample (VIF = 3.362) and Legitimacy and Attitude in the athletes sample (VIF = 5.349 and 4.220, respectively). Because these elevated values concentrate on the conceptually adjacent Legitimacy–Attitude part of the model (see also the discriminant validity discussion below), we interpret them as reflecting construct proximity rather than clear evidence of common method bias. Nevertheless, this finding cautions against fully ruling out CMV, and we therefore complement the VIF assessment with Harman's test and the procedural safeguards described above. Second, we performed Harman's single-factor test on all 32 indicators. The first unrotated factor explained 43.0% of variance among athletes and 41.7% among DCOs, both below the 50% threshold (80). Taken together, the procedural safeguards built into the survey design (separated rating sections, neutral wording, anonymous administration) and these statistical tests indicate that CMB is unlikely to materially affect the structural estimates.

## 6 Results

### 6.1 Evaluation of the measurement model

Most items in our reflective measurement model exceed the commonly accepted loading threshold of 0.708, indicating item reliabilities ≥ 0.50 and thus solid indicator quality [see Table 3 (70)]. A few indicators load between 0.50 and 0.708. We retained them because construct-level quality remained satisfactory. CR (≥ 0.70) and AVE (≥ 0.50) met recommended criteria, and the retained items were theoretically essential for content validity (70, 72, 81). No multicollinearity issues are present, as all Variance Inflation Factor (VIF) values remain below the critical value of 5 (72).

**Table 3:** Measurement items and loadings.

Construct	Item ID	Item	Factor loading	
			Athletes	DCOs
Attitude toward RSS	-	All in all, the introduction of an RSS within the next six months would be..	-	-
	Att1	.. extremely negative to extremely positive	0.937	0.907
	Att2	.. extremely bad to extremely good	0.969	0.941
	Att3	.. extremely harmful to extremely beneficial	0.939	0.906
Benefits	Ben1	I think using the RSS is convenient.	0.794	0.825
	Ben2	Using the RSS will save money.	0.586	0.547
	Ben3	I can save time by using the RSS.	0.825	0.800
	Ben4	Using the RSS enables me to accomplish a doping test more quickly compared to conventional testing.	0.717	0.797
	Ben5	Using the RSS increases my productivity.	0.805	0.853
Legitimacy	Leg1	Using an RSS is fully justified because it protects clean sport.	0.942	0.941
	Leg2	Using an RSS is effective in protecting clean sport.	0.948	0.944
	Leg3	Using an RSS is fair to all athletes.	0.941	0.923
	Leg4	Using an RSS can enhance the equality in all sports and all countries.	0.903	0.922
Performance Risk	PeR1	The Remote Sampling System might not perform well and create problems.	0.811	0.758
	PeR2	The security systems built into the Remote Sampling System are not strong enough to protect my data.	0.675	0.816
	PeR4	Remote Sampling System's servers may not perform well and process information incorrectly.	0.760	0.758
	PeR5	What is the likelihood that there will be something wrong with the performance of the Remote Sampling System or that it will not work properly?	0.911	0.855
Privacy Risk	PrR1	The RSS would collect too much information about a user.	0.942	0.696
	PrR2	I would be concerned about my privacy when using the RSS.	0.931	0.921
	PrR3	I have doubts as to how well my privacy is protected while using the RSS.	0.895	0.909
	PrR4	My personal information would be misused when the RSS is running.	0.890	0.909
	PrR5	My personal information would be accessed by unknown parties when using the RSS.	0.942	0.897
Psychological Risk	PsR1	The thought of using the RSS causes me to experience unnecessary tension.	0.924	0.940
	PsR2	The thought of using the RSS gives me a feeling of unwanted anxiety.	0.959	0.964
	PsR3	I would worry a lot when using the RSS.	0.938	0.925
Transparency	Tra1	I believe the working processes of an RSS would be transparent.	0.944	0.772
	Tra2	I believe the testing authorities will provide me with deep access to how an RSS works.	0.666	0.914
	Tra3	I believe the testing authorities will provide me with in-depth knowledge about operations of the RSS.	0.651	0.925
Trust in RSS	Tru1	The RSS is a very reliable piece of software.	0.759	0.768
	Tru2	The RSS will not fail me.	0.772	0.795
	Tru3	I expect the RSS to be extremely dependable.	0.512	0.747
	Tru5	The RSS has the functionality I need.	0.851	0.886
	Tru6	The RSS has the features required for my tasks.	0.781	0.881

Furthermore, convergent validity across constructs was confirmed, as the AVE for all constructs in both samples exceeded the threshold of 0.50. Discriminant validity was assessed using the Fornell–Larcker criterion, which was fulfilled for both samples. The square root of each construct's AVE was greater than its correlations with any other construct (see Tables 4, 5). Additionally, the Heterotrait-Monotrait (HTMT) ratios of correlations, calculated separately for both subgroups (see Tables 4, 5), are mostly below the critical threshold of 0.90, Most heterotrait-monotrait (HTMT) ratios were below the conservative 0.90 threshold across both samples. The only exception was the Legitimacy–Attitude pair in the athletes sample (HTMT = 0.909). A 95% bootstrap confidence interval (5,000 subsamples) for this HTMT was [0.857, 0.949], excluding the value of 1.0 and thus satisfying the HTMTinference criterion of discriminant validity (78). The point estimate marginally exceeds the conservative 0.90 threshold, reflecting the strong theoretical proximity of legitimacy (a normative evaluation) and attitude (a summative evaluation) in the present mandatory-technology context. For all remaining pairs in both samples, HTMT values were below 0.86, well within accepted thresholds. We complement this assessment with Cohen's f^2^ values for all structural paths [Cohen, 1988 (70)]. Among athletes, the dominant effects are observed for the relationships between Legitimacy and Attitude (f^2^ = 2.909), between Trust and Psychological Risk (f^2^ = 0.547), between Performance Risk and Legitimacy (f^2^ = 0.468) and between Trust and Performance Risk (f^2^ = 0.414). For DCOs, the dominant effects are observed for the relationships between Legitimacy and Attitude (f^2^ = 1.302), between Transparency and Trust (f^2^ = 0.549), between Trust and Psychological Risk (f^2^ = 0.407) and between Trust and Benefits (f^2^ = 0.395). The relationship between Legitimacy and Attitude exceeds conventional Cohen-thresholds in both groups. Because Legitimacy is the sole predictor of Attitude in the model, this f^2^ value mathematically reduces to R^2^/(1−R^2^) and carries no information beyond R^2^ (70). The remaining structural paths range from small to large in effect size, broadly consistent with the significance pattern reported in Table 7. Internal consistency reliability was evaluated using ɑ and CR. Both indicators exceed the threshold of 0.70 in all cases (see Tables 4, 5), indicating that the measurement model demonstrates sufficient internal consistency reliability.

**Table 4:** Athletes’ survey reliability checks, correlations and HTMT.

Construct	ɑ	CR	AVE	Tra	Tru	Ben	PeR	PrR	PsR	Leg	Att
Transparency (Tra)	0.788	0.804	0.586	–	0.351	0.577	0.636	0.511	0.419	0.694	0.605
Trust (Tru)	0.789	0.858	0.553	0.267	–	0.499	0.668	0.503	0.671	0.620	0.652
Benefits (Ben)	0.814	0.864	0.564	0.002	0.403	–	0.565	0.298	0.483	0.572	0.517
Performance Risk (PeR)	0.800	0.871	0.630	0.462	−0.540	−0.461	–	0.765	0.75	0.866	0.786
Privacy Risk (PrR)	0.937	0.952	0.800	−0.520	−0.44	−0.263	0.669	–	0.684	0.483	0.475
Psychological Risk (PsR)	0.935	0.958	0.885	−0.436	−0.586	−0.423	0.648	0.640	–	0.655	0.606
Legitimacy (Leg)	0.951	0.965	0.872	−0.373	0.551	0.507	−0.752	−0.454	−0.617	–	0.909
Attitude (Att)	0.944	0.964	0.899	0.614	0.573	0.459	−0.679	−0.444	−0.568	0.861	–

*α*, Cronbachs alpha; CR, composite reliability; AVE, average variance extracted; correlations in lower triangle; HTMT in upper triangle.

**Table 5:** DCOs’ survey reliability checks, correlations and HTMT.

Construct	ɑ	CR	AVE	Tra	Tru	Ben	PeR	PrR	PsR	Leg	Att
Transparency (Tra)	0.840	0.905	0.762	–	0.679	0.620	0.347	0.225	0.554	0.730	0.662
Trust (Tru)	0.877	0.909	0.668	0.579	–	0.526	0.479	0.352	0.56	0.643	0.591
Benefits (Ben)	0.835	0.879	0.596	0.522	0.447	–	0.289	0.385	0.529	0.603	0.693
Performance Risk (PeR)	0.809	0.875	0.636	−0.282	−0.405	−0.223	–	0.687	0.559	0.531	0.553
Privacy Risk (PrR)	0.918	0.939	0.758	−0.196	−0.316	−0.338	0.535	–	0.572	0.376	0.422
Psychological Risk (PsR)	0.938	0.960	0.899	−0.491	−0.501	−0.472	0.485	0.530	–	0.683	0.784
Legitimacy (Leg)	0.950	0.964	0.869	0.651	0.582	0.541	−0.462	−0.351	−0.645	–	0.809
Attitude (Att)	0.907	0.942	0.843	0.577	0.523	0.607	−0.470	−0.385	−0.723	0.751	–

ɑ, Cronbach's alpha; CR, composite reliability; AVE, average variance extracted; correlations in lower triangle; HTMT in upper triangle.

### 6.2 Mean comparison

The results of the mean comparisons are presented in Table 6.

**Table 6:** Mean comparisons.

Construct	Mean (SD) Athletes	Mean (SD) DCOs	Mean difference	p	Cohen's d	Bonferroni-corrected p
Perceived Transparency	4.336 (1.369)	4.206 (1.571)	−0.13	0.501	0.088	1
Trust in RSS	3.530 (1.225)	3.409 (1.276)	−0.121	0.459	0.097	1
Perceived Benefits	4.061 (1.439)	4.267 (1.367)	0.206	0.257	−0.147	1
**Performance risk**	**4.348** (**1.204)**	**4.862** (**1.404)**	**0**.**514**	**0**.**003**	**−0**.**393**	**0**.**024**
Privacy risk	3.238 (1.635)	3.718 (1.627)	0.48	0.026	−0.291	0.208
Psychological risk	3.497 (1.827)	3.894 (1.946)	0.397	0.109	−0.21	0.872
**Perceived Legitimacy**	**3.712** (**1.915)**	**2.923** (**1.646)**	**−0**.**789**	**0**.**001**	**0**.**442**	**0**.**008**
**Attitude toward RSS**	**2.942** (**1.170)**	**2.364** (**1.142)**	**−0**.**578**	**0**.**001**	**0**.**499**	**0**.**008**

Significant differences in mean values are marked in bold.

The mean comparison results indicate that DCOs perceive significantly higher levels of performance risk associated with the RSS than athletes. In contrast, athletes report significantly higher perceived legitimacy and more favorable attitudes toward the RSS. While the initial *p*-value for privacy risk suggested a difference (*p* = 0.026), this effect did not remain statistically significant after applying the Bonferroni correction (corrected *p* = 0.208). For this and all other variables, no significant differences were observed, indicating a general alignment between the two stakeholder groups.

### 6.3 PLS-SEM and MGA

**Figure 3:**
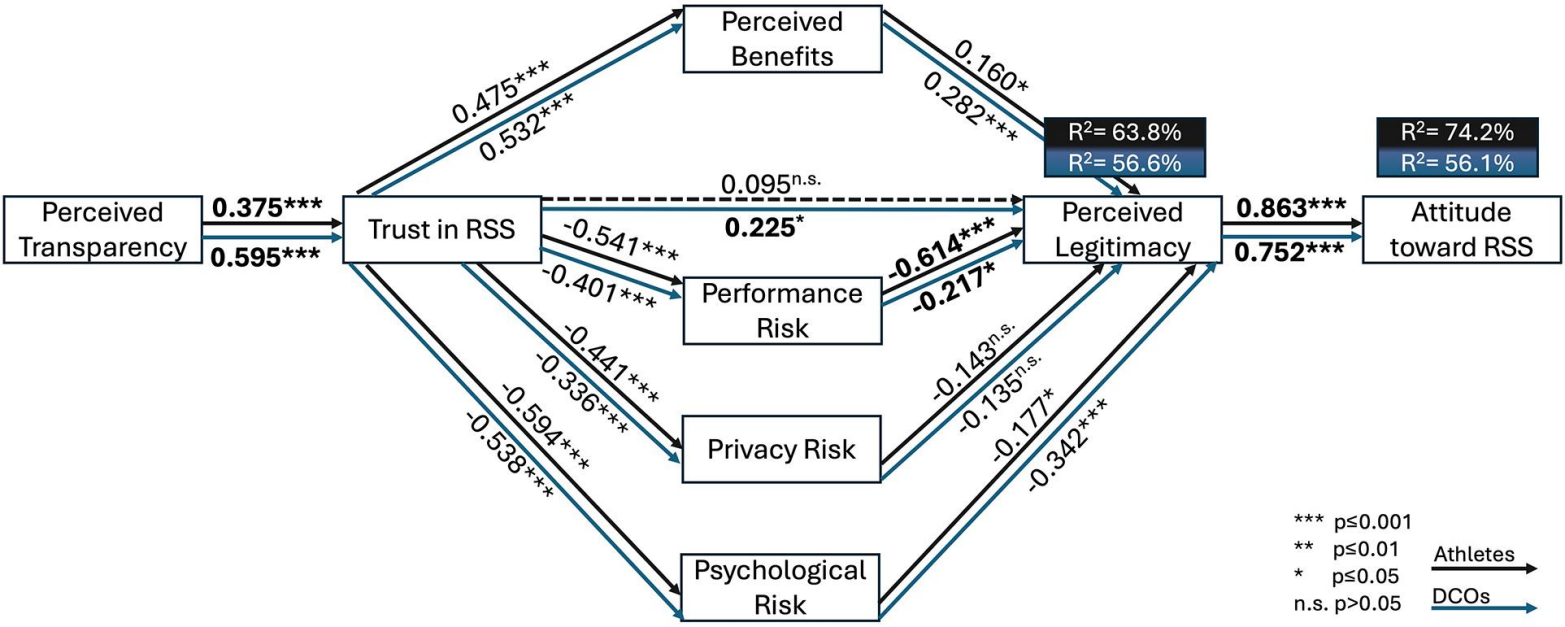
Results of PLS-SEM and MGA.the relationships between variables marked with bold fonts differ significantly between athletes and DCOs, as indicated by MGA.

To test the proposed research model and its hypotheses, we employed PLS-SEM. The model was estimated independently for the athlete and DCO samples, followed by an MGA to identify statistically significant differences in the path coefficients between the two groups. The detailed results of these analyses are presented in Figure 3 and Table 7.

**Table 7:** Results of the PLS-SEM and multigroup analysis.

Hypothesis	Athletes		DCOs		Hypothesis assessment	MGA (Differences)	
	*β*	p	β	p		ß	p
H1: Transparency → Trust	**0**.**375**	**<0**.**001**	**0**.**595**	**<0**.**001**	Supported	**−0**.**220**	**0**.**035**
H2: Trust → Benefits	**0**.**475**	**<0**.**001**	**0**.**532**	**<0**.**001**	Supported	−0.057	0.498
H3a: Trust → PerformanceR	**−0**.**541**	**<0**.**001**	**−0**.**401**	**<0**.**001**	Supported	−0.140	0.259
H3b: Trust → PrivacyR	**−0**.**441**	**<0**.**001**	**−0**.**336**	**0**.**001**	Supported	−0.105	0.366
H3c: Trust → PsychologicalR	**−0**.**594**	**<0**.**001**	**−0**.**538**	**<0**.**001**	Supported	−0.057	0.495
H4: Trust → Legitimacy	0.095	0.114	**0**.**225**	**0**.**011**	Partially supported	−0.130	0.225
H5: Benefits → Legitimacy	**0**.**160**	**0**.**024**	**0**.**282**	**0**.**001**	Supported	−0.122	0.280
H6a: PerformanceR → Legitimacy	**−0**.**614**	**<0**.**001**	**−0**.**217**	**0**.**033**	Supported	**−0**.**397**	**0**.**005**
H6b: PrivacyR → Legitimacy	0.143	0.062	0.135	0.116	Rejected	0.008	0.934
H6c: PsychologicalR → Legitimacy	**−0**.**177**	**0**.**027**	**−0**.**342**	**<0**.**001**	Supported	0.164	0.193
H7: Legitimacy → Attitude	**0**.**863**	**<0**.**001**	**0**.**752**	**<0**.**001**	Supported	**0**.**111**	**0**.**020**

R, risk, β, standardized effect size, significant effects at the 5% level (*p* < 0.05) are marked in bold.

Starting with PLS-SEM results, the model demonstrated substantial explanatory power for the athlete sample. It accounted for 63.8% of the variance in Perceived Legitimacy and 74.2% of the variance in Attitude toward RSS (R^2^ adjusted: 0.638 and 0.742, respectively).

As hypothesized, Perceived Transparency had a significant positive effect on Trust in RSS (*β* = 0.375, *p* < 0.001), supporting H1. In turn, Trust was positively associated with Perceived Benefits (*β* = 0.475, *p* < 0.001; H2 supported) and negatively associated with all three risk dimensions: Performance Risk (*β* = −0.541, *p* < 0.001; H3a supported), Privacy Risk (*β* = −0.441, *p* < 0.001; H3b supported), and Psychological Risk (*β* = −0.594, *p* < 0.001; H3c supported). Regarding the antecedents of Perceived Legitimacy, Perceived Benefits exerted a significant positive influence (*β* = 0.160, *p* = .024), while Performance Risk (*β* = −0.614, *p* < 0.001) and Psychological Risk (*β* = −0.177, *p* = 0.027) had significant negative effects, supporting H5, H6a, and H6c, respectively. For athletes, the direct path from Trust to Perceived Legitimacy was not significant (*β* = 0.095, *p* = 0.114). Privacy Risk also did not show a significant direct effect on Legitimacy (*β* = 0.143, *p* = 0.062). H4 is therefore not supported for athletes, while H6b is rejected for both groups. However, given the strong indirect effects of Trust on Legitimacy reported below, we interpret H4 for athletes as a case of full mediation rather than as a null effect. Finally, Perceived Legitimacy was a very strong positive predictor of Attitude toward RSS (*β* = 0.863, *p* < 0.001), providing strong support for H7.

For the DCO sample, the model also showed strong predictive relevance, explaining 56.6% of the variance in Perceived Legitimacy and 56.1% of the variance in Attitude (R^2^ adjusted: 0.556 and 0.561, respectively). The pattern of results was largely consistent with the athlete sample. H1, H2, and H3a-c were all supported, indicating that Transparency significantly predicts Trust, which in turn predicts higher Benefits and lower perceptions of Performance, Privacy, and Psychological Risk. Similar to the athlete model, Perceived Benefits (*β* = 0.282, *p* = 0.001), Performance Risk (*β* = −0.217, *p* = 0.033), and Psychological Risk (*β* = −0.342, *p* < 0.001) were significant predictors of Perceived Legitimacy, supporting H5, H6a, and H6c. The path from Trust to Perceived Legitimacy was modestly significant (*β* = 0.225, *p* = 0.011), supporting H4 for DCOs. The path from Privacy Risk to Perceived Legitimacy was again non-significant, leading to the rejection of H6b. Lastly, Perceived Legitimacy strongly and positively influenced Attitude (*β* = 0.752, *p* < 0.001), supporting H7.

To examine the structural role of trust in shaping legitimacy, we additionally examined indirect and total effects with bias-corrected bootstrap confidence intervals (5,000 subsamples). Although the direct relationship between Trust and Legitimacy is non-significant for athletes (*β* = 0.095, *p* = 0.114) and only modestly significant for DCOs (*β* = 0.225, *p* = 0.011), the corresponding specific indirect effects operating through the benefit-risk evaluations are substantial. In the athletes sample, Trust exerts significant indirect effects on Legitimacy via Performance Risk (*β* = 0.332, *p* < 0.001), Psychological Risk (*β* = 0.105, *p* = 0.028) and Benefits (*β* = 0.076, *p* = 0.041), while the indirect path through Privacy Risk is non-significant (*β* = −0.063, *p* = 0.078; the sign reflects the counter-hypothesized positive direct effect of Privacy Risk on Legitimacy). Among DCOs, the indirect effects through Psychological Risk (*β* = 0.184, *p* = 0.001), Benefits (*β* = 0.150, *p* = 0.006) and Performance Risk (*β* = 0.087, *p* = 0.068) carry the largest weight. The total effect of Trust on Legitimacy is significant and substantial in both groups (athletes *β* = 0.546, *p* < 0.001; DCOs *β* = 0.601, *p* < 0.001). These results indicate that Trust shapes Legitimacy primarily through users’ benefit-risk evaluations rather than directly, with risks (especially Performance Risk for athletes) acting as the dominant mediating pathway. We therefore interpret H4 as fully mediated for athletes and partially mediated for DCOs rather than rejected.

The MGA was conducted to formally test for differences in the structural path estimates between athletes and DCOs. The analysis revealed three statistically significant differences. First, the positive influence of Perceived Transparency on Trust in RSS (H1) was significantly stronger for DCOs (*β* = 0.595) than for athletes (*β* = 0.375, *p* = 0.035). Second, the negative effect of Performance Risk on Perceived Legitimacy (H6a) was significantly more pronounced for athletes (*β* = −0.614) compared to DCOs (*β* = −0.217, *p* = 0.005). Third, the relationship between Perceived Legitimacy and Attitude toward RSS (H7) was also significantly stronger for athletes (*β* = 0.863) than for DCOs (*β* = 0.752, *p* = 0.020). No other path coefficients differed significantly between the two groups.

## 7 Discussion

### 7.1 Differences in athletes’ and DCOs’ perceptions about remote sampling

Compared with athletes, DCOs perceived significantly higher performance risk associated with the RSS. This pattern is consistent with DCOs’ specialized role as custodians of procedural integrity (82). Their deeper familiarity with regulated testing protocols, like chain-of-custody requirements, and potential failure points likely makes them more critical regarding the functional demands that an RSS must meet to be considered acceptable. This suggests that DCOs pay particular attention to the functional reliability and technical robustness of the RSS and the associated procedures. Additionally, it shall be noted that differences in user age can influence the perception of new technologies, with older users reporting higher risk perceptions (83, 84). As DCOs are, on average, considerably older than athletes in the investigated samples, this effect may contribute further to the abovementioned role differences.

Athletes report significantly higher perceived legitimacy and more favorable attitudes toward the RSS compared to DCOs. One plausible explanation is that DCOs, by virtue of their professional commitment to the existing testing regime, may be more attached to the traditional, in-person procedures and more cautious toward a technological innovation that alters established routines, potentially even threatening their current job roles. In contrast, athletes have no choice but to comply with the testing system imposed on them, which, in its current configuration, is often experienced as stressful and burdensome (85, 86). For athletes who perceive the current system as problematic, a technological innovation such as the introduction of an RSS can be interpreted as a meaningful and legitimate improvement. This likely reflects a greater openness to digitally supported testing methods and may be linked to anticipated gains in convenience, flexibility, and perceived procedural fairness. These more positive perceptions among athletes represent a strategic opportunity to support system rollout and to strengthen athletes’ broader perceptions of anti-doping legitimacy and acceptance (87).

For all other variables, no significant differences were observed, pointing to a broad alignment between the two stakeholder groups in their evaluations of RSS. This convergence suggests that, on average, athletes and DCOs form similar perceptions of many key aspects of the system.

### 7.2 Key influences shaping athletes and DCOs legitimacy perception and attitude

The influence of performance risk on perceived legitimacy of RSS was significantly stronger in athletes compared to DCOs. This indicates that even though DCOs perceive a higher level of performance risks, athletes are especially sensitive to the system's functional reliability when considering its legitimacy in the anti-doping context (87, 88). Since performance risk refers to the possibility of the RSS failing to perform as intended (53), this finding suggests that technical reliability and secure, standardized processes are central to athletes’ perceptions of the legitimacy of remote sampling in anti-doping. Moreover, the significantly stronger influence of Perceived Legitimacy on Attitude among athletes emphasizes the central role of legitimacy in shaping overall user assessments of the RSS (89).

This finding implies that even in a mandatory use context, where opting out is not possible, user acceptance cannot be taken for granted. Athletes with negative attitudes may disengage, resist, or undermine the system, making it imperative to build legitimacy through transparent communication, equitable enforcement, and stakeholder engagement

The mediation analysis introduced in the Results section adds an important nuance to these direct-effect comparisons. Although the direct relationship between Trust and Legitimacy is non-significant for athletes and only modestly significant for DCOs, the corresponding total effects of Trust on Legitimacy are substantial in both groups. Trust thus operates predominantly through the benefit-risk evaluations rather than directly. The dominant mediating pathways, however, differ between the two groups. For athletes, the strongest specific indirect effect runs through Performance Risk, consistent with their heightened sensitivity to functional reliability identified above. For DCOs, the strongest indirect effects run through Psychological Risk and Benefits, reflecting their dual concern with the procedural burden of an unfamiliar technology and the operational benefits it can offer. These role-specific mediation patterns reinforce the interpretation that acceptance of mandatory anti-doping technologies emerges through different psychological routes for those administering and those subject to the testing regime, even when the overall importance of trust is comparable.

## 8 Contributions and implications

### 8.1 Contributions to research

This study makes several contributions to research. First, established technology acceptance models in information systems research treat adoption as voluntary, centering on perceived usefulness and ease of use (90) which are synthesized into theories on technology acceptance [e.g., UTAUT (91)]. Mandatory adoption redirects user concerns from utility toward fairness, procedural justice, and legitimacy (92). This work integrates the concept of legitimacy into the decomposed and contextualized EVF provided by Stoffers et al. (11). Our results are consistent with the idea that perceived legitimacy occupies a central position in structuring acceptance of new technologies in a mandatory-use, high-stakes context. Here, overall attitude functions as an integrative summary judgment of the users’ evaluations of benefits and risks towards RSS introduction, and the relationship of benefit and risk evaluations towards attitude is structured through the users’ legitimacy perception forming an initial cognitive appraisal that subsequently shapes the overall attitude towards the mandatory system. The resulting framework transfers to comparable sectors such as e-health [see e.g. (93)], employee monitoring [see e.g. (94)], and e-government [see e.g. (51)].

Second, this study shows the value of differentiated stakeholder perspectives. Rather than treating “users” as a homogeneous group, our findings indicate that stakeholder roles and characteristics shape how specific factors are weighted. Transparency exerts a stronger influence on trust among DCOs, whereas performance risk more strongly undermines perceived legitimacy among athletes, and legitimacy in turn has a stronger association with athletes’ attitudes. These role-specific patterns demonstrate that acceptance mechanisms can differ meaningfully between stakeholder groups who are embedded in the same system but occupy distinct positions and responsibilities. This is consistent with evidence that compliance-role occupants (i.e., DCOs) treat procedural clarity and transparency as a primary signal of institutional integrity [see e.g. (40)]. Performance risk more strongly undermines legitimacy among affected subjects (i.e., athletes), mirroring organizational justice research showing that subjects of mandatory oversight weight outcome-related risks more heavily than those administering the system (95). Such role differentiations can be utilized in related research domains on e.g., digital proctoring, where instructors and students evaluate surveillance technology through different fairness lenses [see e.g. (96)].

Third, the study refines the role of trust within EVF-based technology acceptance models. In our structural model, trust is closely linked to higher perceived benefits and lower perceived performance, privacy, and psychological risks, but exerts only a modest residual direct effect on legitimacy once these evaluations are accounted for (non-significant for athletes; *β* = 0.225, *p* = 0.011 for DCOs). This suggests that, in a mandatory and control-oriented context such as anti-doping, trust operates predominantly by shaping how users interpret the potential upsides and downsides of a system. This mediation is complete for athletes, whereas a modest direct effect of trust on legitimacy remains for DCOs alongside the dominant indirect pathways. Also, this study adds nuance to the risk component of EVF by unpacking how different risk dimensions relate to legitimacy in a surveillance-like setting. Performance and psychological risks significantly erode perceived legitimacy, whereas privacy risk does not show a significant effect once other risks are included. This pattern suggests that, in the context of remote anti-doping testing, concerns about whether the system functions reliably and whether it increases psychological burden are more consequential for legitimacy than abstract worries about privacy. Such a differentiated risk profile highlights the importance of context (97) when applying and extending valence-based models of technology evaluation.

Fourth, this study acts as a bridge between research on technology acceptance, especially in mandatory use contexts [see e.g, (15, 51)] and the field of sports management and sports digitalization research. It demonstrates the utility of information systems theory [specifically, the EVF (17)] with sports management and digitalization research as a domain where socio-technical challenges surrounding e.g., digitalization and athlete monitoring have thus far been examined without the systematic application of established acceptance frameworks (see e.g., investigations on the trust of athletes in anti-doping interventions (6), interview studies on athletes privacy concerns regarding the ADAMS whereabouts system (31, 98). To expand on this studies rich contextual insight, modeling the underlying acceptance mechanisms is a gap that technology acceptance research can address, as underlined in this study.

Finally, this study also contributes to the social science research agenda on anti-doping, which emphasizes the need to better understand athletes’ experiences with key anti-doping practices, such as testing (99). This is achieved by examining athletes’ and DCOs’ perceptions of remote sampling as a technological innovation in testing procedures.

### 8.2 Implications for practice

To derive practical implications, the current regulatory environment must be considered, as it defines the scope for implementing remote sampling in anti-doping practice. WADA regulations, including the 2027 WADA Code (100) and the new International Standard for Testing [IST (2)], continue to permit remote sampling procedures only in specific circumstances, such as a pandemic. Further, these rules define remote testing as a “partially virtual” hybrid system, in which DCOs still conduct in-person notification of athletes and handle transport of collected samples. Within this framework, ADOs are responsible for selecting a suitable IT system and educating athletes and DCOs on its use, with WADA providing basic guidance for these processes. While current regulations do not accommodate a fully virtual RSS or the use of DBS sampling in a virtual setting, our study offers insights that are particularly relevant for ADOs and technology providers planning pilot trials or future developments aimed at supplementing traditional testing in pursuit of detection and deterrence. In this context, we highlight user perceptions that appear critical for technology implementation that is perceived as legitimate by both DCOs and athletes.

A central finding is that establishing trust among both athletes and DCOs is essential, and can be effectively strengthened through transparency. The analysis indicates that transparency has a notably stronger effect on trust for DCOs, underscoring the need for clear information about system functions, responsibilities, and safeguards. Transparent communication regarding procedures, rules, and data management is helpful and voiced in the complementary qualitative study interviewing athletes and DCOs. System developers should therefore integrate features that promote transparency, such as verifiable audit trails, thorough documentation, and real-time monitoring capabilities tailored to the DCO user group. ADOs, in turn, should ensure that DCOs are not only trained in using the system but also equipped to explain and justify its functioning to athletes.

Furthermore, our results show that DCOs perceive significantly higher performance risks associated with remote sampling than athletes. This aligns with their role as custodians of procedural integrity and chain-of-custody compliance. For athletes, doubts about system functionality or fears of malfunction diminish their perceived legitimacy of the technology, which is particularly critical given the profound career implications of anti-doping tests for athletes. To address these concerns, technology providers should prioritize system reliability, robustness, and clear error-handling. Regulators and ADOs should mandate and implement explicit fallback protocols that protect athletes from the consequences of unforeseeable technical failures (for example, system outages or connectivity problems), and these safeguards should be communicated clearly to both athletes and DCOs. This can alleviate concerns that technical problems might unfairly affect test outcomes or disciplinary decisions.

While WADA regulations rightly emphasize privacy and data protection measures in the context of virtual sample collection, our results indicate that privacy risks are not the primary drivers of users’ legitimacy perceptions. Instead, psychological risks and performance-related fears more strongly erode legitimacy. Practical measures should therefore extend beyond data protection compliance. Educational initiatives for athletes and DCOs should focus on mitigating stress, anxiety, and perceived vulnerability associated with a new, high-stakes procedure, as well as clarifying how the system ensures correct and reliable procedures. This could include hands-on demonstrations, clear explanations of verification steps, and channels for feedback and concerns. The goal is to build confidence and acceptance, particularly among athletes, by making the procedure feel predictable, fair, and manageable.

Although the current regulatory landscape restricts the broad application of remote sampling, our findings support the value of further pilot applications under controlled conditions. For successful development and broader rollout of RSS, strategies that strengthen user trust, guarantee system reliability, and address the specific psychological and performance-related concerns of both athletes and DCOs are crucial. Addressing these human factors is at least as important as resolving technological and regulatory issues and will be central to the evolution of anti-doping practices involving remote sampling.

### 8.3 Limitations and future research

Our study is not without limitations, which open several avenues for future investigation. The low level of direct experience with RSS among our participants represents a key constraint, as RSS has not yet been applied widely in anti-doping practice. With only roughly 5% of both athletes and DCOs having participated in actual remote testing pilots, our findings reflect anticipated perceptions rather than judgments based on personal experience. Participants evaluated RSS based on a described remote sampling procedure and associated study materials rather than a fully deployed and diversified set of live systems. Their perceptions thus refer to a specific scenario and prototype-like representation of RSS. In practice, design choices such as sampling medium, user interface, communication channels, or support processes may meaningfully alter perceived benefits, risks, and legitimacy. While this provides valuable insight into initial barriers and drivers of adoption, future research should track how these perceptions evolve as stakeholders gain hands-on experience. It is plausible that performance risks loom larger in imagination than in everyday practice, or conversely, that unforeseen operational friction points only emerge after repeated use. Technology acceptance research has consistently demonstrated that pre-adoption beliefs and post-adoption evaluations diverge once direct experience is gained, with anticipated risks often exceeding what users actually encounter in practice (68). A comparable dynamic was observed in the adoption of COVID-19 contact tracing apps, where initial resistance driven by strong privacy and surveillance concerns gave way to increased trust and acceptance following actual use (41).

A second limitation concerns the composition and scope of our sample. The study focuses on athletes and DCOs from specific organizational and sporting contexts, which may differ from other regions, sports, or anti-doping systems in terms of culture, regulatory environment, and prior exposure to digital tools. As a result, the generalizability of our findings is limited. Future research should replicate and extend this work across a broader range of sports, competition levels, and national anti-doping organizations, as well as in contexts with different histories of doping and testing intensity, to examine whether the identified patterns hold or vary across settings.

Beyond the sampling concerns discussed above, a related limitation pertains to contextual factors that we could not formally test in the present design. Prior research has identified several factors that plausibly shape RSS perceptions, including sport type (team vs. individual), competitive tier (e.g., Registered Testing Pool membership), national or cultural context, prior anti-doping or RSS experience, and gender- or culture-specific concerns regarding the intrusiveness of traditional in-person observation (57, 64, 98, 101). Traditional direct-observation testing may disproportionately disadvantage female athletes and may conflict with cultural or religious norms in some settings, suggesting that perceptions of RSS, including the strength of the legitimacy-to-attitude relationship, could be moderated by the starting point, that is, by how intrusive the traditional alternative is perceived to be. The available dataset contains operationalizable proxies for each of these factors. However, we did not estimate multi-group comparisons across these contextual factors because the resulting subgroup sample sizes (e.g., *n* = 26 athletes in the Registered Testing Pool; *n* = 19 in the National Pool; *n* < 15 with any direct RSS experience) fall well below the minimum required for adequate statistical power under our *a priori* assumptions [see the power analysis in the Methods section (72)]. Reporting subgroup PLS-MGA estimates on such small subsamples would be statistically unreliable and could be misleading. We therefore restrict the present study to the two principal stakeholder groups (athletes and DCOs) and call for future research using larger, more balanced samples to test whether sport type, competitive tier, regulatory regime, and direct RSS exposure systematically moderate the relationships identified here.

A further limitation concerns causal inference. Although the structural model specifies theoretically derived directional paths, the cross-sectional survey design does not allow causal conclusions. The reported coefficients should therefore be interpreted as associations consistent with the proposed theoretical model rather than as evidence that transparency, trust, benefits, or risks causally produce legitimacy or attitudes. Moreover, the study measures perceptions rather than behavioral outcomes such as actual compliance behavior, sample-collection success rates, error rates, or post-implementation use experiences. Future research should therefore combine longitudinal perception data with behavioral and operational implementation data.

Moreover, the non-significant impact of privacy risk on legitimacy warrants deeper qualitative exploration. It remains unclear whether this finding reflects a genuine lack of concern about privacy in the anti-doping context, or a trade-off in which users prioritize functional reliability and psychological safety over data-related worries. Research suggests that athletes subject to intensive whereabouts requirements often rationalize privacy intrusions as a condition of elite sport, effectively normalizing continuous monitoring (57, 98). Studies examining athlete knowledge and assessment of the ADAMS system found a context-specific privacy calculus in which the clean sport mandate functions as a legitimizing frame that dampens privacy salience (85). Here, clean athletes accept surveillance burdens they view as necessary costs of competing in a trustworthy system.

## 9 Conclusion

This study advances understanding of how RSS can become a legitimate component of anti-doping practice. Conceptually, we extend a contextualized EVF by positioning perceived legitimacy as a central mechanism that connects users’ benefit–risk evaluations with their overall stance toward RSS in a mandatory, high-stakes environment. By differentiating athletes and DCOs, we show that acceptance mechanisms are role-specific and that trust operates primarily through its influence on how risks and benefits are interpreted, with a modest additional direct effect on legitimacy among DCOs. We also nuance the risk component of valence-based models by demonstrating that, in surveillance-like settings, concerns about functional reliability and psychological burden can outweigh abstract privacy concerns.

For anti-doping organizations and technology providers, RSS introduction could fruitfully be approached as a change-management process rather than a purely technical rollout. Systems must be technically robust, provide clear error-handling and fallback protocols, and embed transparent processes and auditability to satisfy DCOs’ custodial responsibilities. At the same time, user-centered design, intuitive interfaces, realistic training, and clear communication about fairness and data handling are needed to reduce athletes’ stress and strengthen their perceptions of legitimacy. Ultimately, successful RSS implementation depends on building systems that are not only compliant with regulations but also trusted by those who operate and are subjected to them.

## Data Availability

The raw data supporting the conclusions of this article will be made available by the authors, without undue reservation.
